# The association between perceived social support and internet addiction among Chinese civil aviation male flight students: chain mediating effects of positive coping strategies and basic psychological need satisfaction

**DOI:** 10.3389/fpsyg.2026.1728338

**Published:** 2026-04-10

**Authors:** Enliang Hu, Haozhe Wang, Jiayi Yao, Wenjia Chen, Mingyu Liao, Shiguan Jia, Qinghua Zhang

**Affiliations:** 1 Civil Aviation Flight Academy of China, Deyang, China; 2 School of Physical Education, China University of Mining and Technology, Xuzhou, China

**Keywords:** basic psychological need satisfaction, flight students, internet addiction, perceived social support, positive coping strategies

## Abstract

**Background:**

This study aimed to explore the mechanisms underlying the relationship between perceived social support and internet addiction among Chinese civil aviation flight students among Chinese civil aviation flight students, with a focus on examining the chain mediating role of positive coping strategies and basic psychological need satisfaction in this relationship.

**Methods:**

Employing a cross-sectional design and cluster sampling method, 1,235 male flight students from the Civil Aviation Flight University of China (mean age 18.80 ± 0.78 years) were surveyed using questionnaires. Data were collected using the Perceived Social Support Scale (PSSS), Chinese Internet Addiction Scale-Revised (CIAS-R), Simplified Coping Style Questionnaire (SCSQ), and Basic Need Satisfaction in General Scale (BNSG-S). Chain mediation analysis was conducted using SPSS 26.0 and PROCESS macro Model 6.

**Results:**

Perceived social support was significantly and negatively associated with internet addiction among flight students (*B* = −0.36, *p* < 0.01). Both positive coping strategies and basic psychological need satisfaction played significant mediating roles. The mediation effect of positive coping strategies was −0.15, while the mediation effect of basic psychological need satisfaction was 0.03. The chain mediation pathway was also significant. The total indirect effect was −0.113, accounting for 31.4% of the total effect.

**Conclusion:**

Perceived social support is associated with internet addiction among flight students through both independent and chain mediation pathways of positive coping strategies and basic psychological need satisfaction. These findings provide theoretical foundations and practical guidance for developing internet addiction prevention and intervention systems for flight students.

## 1 Introduction

With the profound development of the digital age, internet addiction has emerged as a prominent issue affecting the psychological health of young populations (Marin et al., 2021). Characterized as uncontrolled internet use behavior without the involvement of addictive substances (Chadha et al., 2024; Li et al., 2016), internet addiction has reached a detection rate of 11% among Chinese university students, significantly higher than the international average (Shao et al., 2018). Notably, civil aviation flight students, as a specialized occupational group bearing significant aviation safety responsibilities, face unique training environments characterized by enclosed management, high elimination rates, and continuous assessment pressure (Yu et al., 2022). Unlike general university students who have access to open campuses and diverse offline recreational outlets, flight students’ physical mobility and offline social opportunities are strictly limited during training. Consequently, the internet often becomes their primary accessible outlet for stress relief. This uniquely confined social ecology makes them particularly vulnerable to internet addiction. Furthermore, recent literature highlights the complex dynamics between occupational stress, coping, and digital addiction in specialized populations (Wan et al., 2022). For instance, studies demonstrate that emotion regulation and social support critically mitigate job burnout in young nurses, while social support significantly buffers mobile phone addiction among teenagers (Luo et al., 2025; Dong et al., 2024; Dong et al., 2023). Similarly, positive coping styles have been shown to enhance psychological capital and reduce maladaptive behaviors in high-pressure educational settings (Qiu et al., 2025). Furthermore, internet addiction in this population can lead to sleep deprivation, attention deficits and decreased situational awareness. Even minor cognitive impairments can directly translate into critical operational errors, threatening flight safety (Jia et al., 2025; Huang et al., 2022). However, research on the psychological mechanisms of internet addiction in this specialized population remains a significant gap.

Perceived social support represents an individual’s subjective evaluation of support received from family, peers, and significant others (Acoba, 2024). Existing research demonstrates a significant negative correlation between perceived social support and internet addiction among university students (Naseri et al., 2015). According to the compensatory internet use theory (Hu and Huang, 2024), when real-world environments fail to satisfy individuals’ psychological needs, they may turn to the internet seeking alternative satisfaction (Deutrom et al., 2021). However, the internal mechanisms by which perceived social support influences internet addiction remain unclear, particularly within high-stress occupational groups such as flight students.

From a theoretical perspective, perceived social support may influence internet addiction through multiple pathways. First, coping represents the process by which individuals make behavioral and cognitive efforts to reduce the impact of stressful situations (Riedel et al., 2021). Coping strategies serve as key mechanisms for individuals to regulate stress responses and psychological health under stressful circumstances (Palamarchuk and Vaillancourt, 2021). Social support may promote individuals’ adoption of positive coping strategies (Calhoun et al., 2022), thereby reducing dependence on internet use. Second, self-determination theory posits that basic psychological needs represent innate, intrinsic, and essential psychological requirements that are crucial for individuals’ psychological healthy development, integration, and well-being. Civil aviation flight students, within strictly standardized training systems, often face constraints on autonomous decision-making, competence anxiety, and fluctuations in sense of belonging among their basic psychological needs. When these basic psychological needs remain unsatisfied over extended periods, individuals may be driven to seek compensatory satisfaction in cyberspace. Existing research indicates that social support may help satisfy individuals’ basic psychological needs, reducing their motivation to seek compensation in virtual spaces (Bregenzer and Jimenez, 2021; Chen et al., 2025). More importantly, these factors may exhibit progressive relationships—positive coping strategies may enhance basic psychological need satisfaction, forming a continuous influence pathway. However, whether these potential mechanisms hold true within the flight student population requires empirical validation.

**Figure 1:**
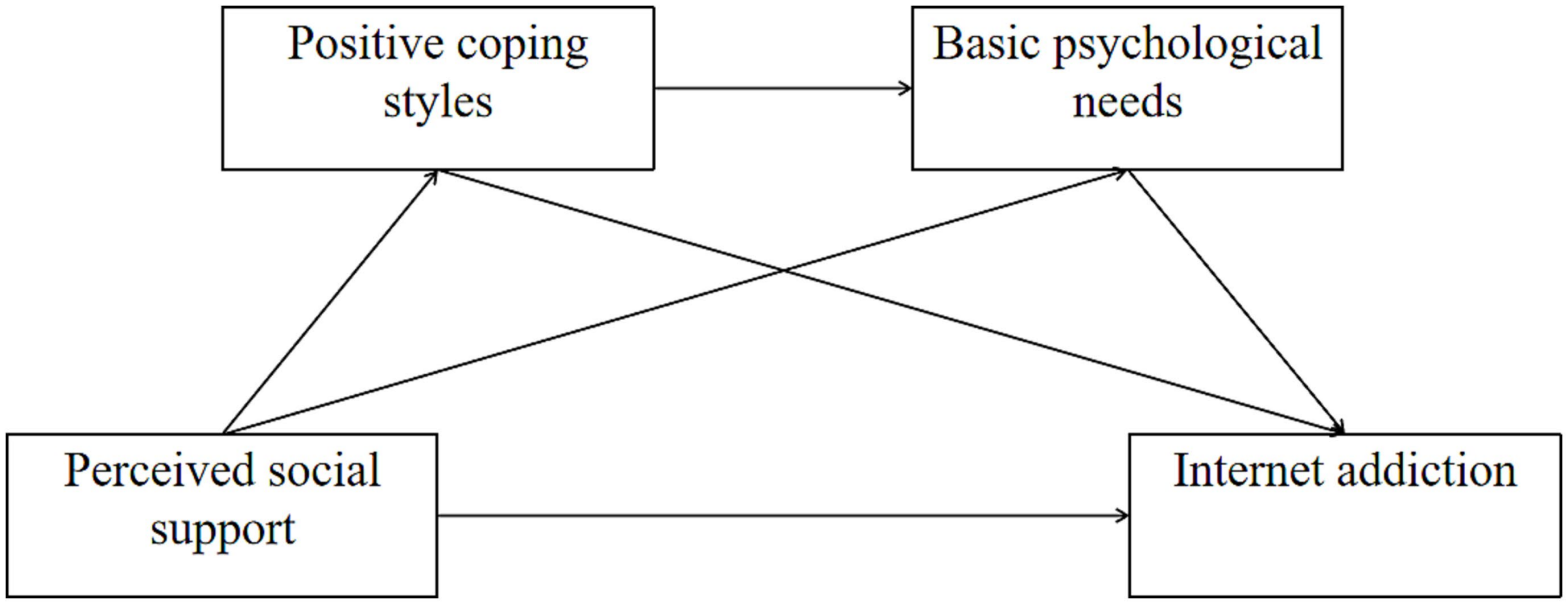
Research hypothesis model.

Integrating these perspectives, this study constructs a comprehensive theoretical framework based on the Compensatory Internet Use Theory, the transactional model of stress and coping, and Self-Determination Theory. We posit that perceived social support acts as a crucial external resource that activates internal positive coping strategies. Within this framework, positive coping styles not only manage stress but actively restore a sense of “environmental control.” By employing active problem-solving or cognitive reframing, flight students can mitigate the unpredictability of high-elimination training. This restored sense of control directly facilitates their basic psychological needs, enhancing their feelings of competence and autonomy in a highly restricted environment, thereby reducing the drive for compensatory internet use. Therefore, this study focuses on civil aviation flight students to explore the mechanisms by which perceived social support influences internet addiction, with particular emphasis on examining the roles of positive coping strategies and basic psychological need satisfaction in this relationship, constructing a more comprehensive chain mediation model (Figure 1). This research not only helps fill the theoretical gap in internet addiction research among specialized occupational groups but also provides scientific evidence for developing targeted psychological health intervention strategies. Based on the above analysis, this study proposes the following hypotheses:

*H1*: Perceived social support is negatively associated with internet addiction among civil aviation flight students.

*H2*: Positive coping strategies mediate the relationship between perceived social support and internet addiction.

*H3*: Basic psychological need satisfaction mediates the relationship between perceived social support and internet addiction.

*H4*: Positive coping strategies and basic psychological need satisfaction exhibit a chain mediation effect in the relationship between perceived social support and internet addiction, whereby perceived social support is related to basic psychological need satisfaction through positive coping strategies, which is subsequently associated with internet addiction.

## 2 Study objects and methods

### 2.1 Participants and procedures

This study employed a cross-sectional design. Considering the training model and management characteristics of flight students, cluster sampling by class was utilized. The specific procedure was as follows: (1) First, a comprehensive list of all flight student classes was obtained, including all teaching classes from first and second years; (2) Simple random sampling was used to randomly select participating classes by grade level; (3) Questionnaire surveys were conducted with all students in the selected classes. During the survey period (December 24, 2024), the overall distribution of flight students at the Civil Aviation Flight University of China was: 1,487 first-year students (56.9%) and 1,126 s-year students (43.1%), totaling 2,613 students. Ultimately, data from 1,235 flight students were included in the analysis. All participants were male, with a mean age of 18.80 ± 0.78 years. Among the participants, 727 were first-year students (58.87%) and 508 were second-year students (41.13%). Chi-square goodness-of-fit test results showed no significant difference between the research sample’s grade distribution and the overall grade distribution (*χ*^2^ = 1.95, df = 1, *p* = 0.16), indicating good representativeness of the research sample in terms of grade composition.

Data were collected through online questionnaires distributed to participating students, containing measurement items for physical activity, internet addiction, perceived social support, and psychological resilience. Inclusion criteria included: (1) currently enrolled students at the Civil Aviation Flight University of China; (2) voluntary participation in the survey. Exclusion criteria included: (1) Completion time screening—based on questionnaire length (75 items) and normal reading speed, the reasonable minimum completion time was set at 300 s (5 min) to prevent timeout and careless responses; (2) Data completeness screening—exclusion of data with missing key variables. After applying these quality control measures, 74 responses were excluded (71 with completion time <300 s, 3 with incomplete data), yielding 1,235 valid responses with a final valid response rate of 94.34%.

### 2.2 Research tools

(1) Perceived Social Support Scale (PSSS)

The Perceived Social Support Scale (PSSS), originally developed by Zimet et al. (1988) and revised by Jiang (2009) (Chen et al., 2016) for the Chinese population, was used to measure the perceived social support levels of flight students. The scale comprises three dimensions: family support (4 items), friend support (4 items), and significant other support (4 items), totaling 12 items. A 7-point Likert scale is employed (1 = strongly disagree, 7 = strongly agree), with total scores ranging from 12 to 84 points. Higher scores indicate higher levels of perceived social support. The scale has demonstrated good reliability and validity among Chinese university students (Tian et al., 2025; Fan, 2020). In this study, the overall Cronbach’s *α* coefficient for the scale was 0.98, with α coefficients for the three dimensions being: family support 0.93, friend support 0.95, and significant other support 0.96. Confirmatory factor analysis showed good structural validity: RMR = 0.04, PGFI = 0.52, PNFI = 0.73, PCFI = 0.73, SRMR = 0.04.

(2) Revised Chinese Internet Addiction Scale (CIAS-R)

The Revised Chinese Internet Addiction Scale (CIAS-R), originally developed by Chen et al. (2003) and revised by Bai and Fan (2005) for the Chinese population, was used to assess the degree of internet addiction among flight students. The scale includes four dimensions: compulsive use and withdrawal symptoms (Sym-C), tolerance symptoms (Sym-T), interpersonal and health-related problems (RP-IH), and time management problems (RP-TM), comprising 19 items in total. A 4-point Likert scale is used (1 = does not apply at all, 4 = applies very much), with total scores ranging from 19 to 76 points. Higher scores indicate more severe internet addiction. A cutoff score of 53 points is used, with scores ≥53 indicating internet addiction. In this study, the overall Cronbach’s *α* coefficient for the scale was 0.97, with all dimensional α coefficients above 0.85. Confirmatory factor analysis indicated good model fit: RMR = 0.03, PGFI = 0.59, PNFI = 0.77, PCFI = 0.78, SRMR = 0.06.

(3) Simplified Coping Style Questionnaire (SCSQ)

The positive coping dimension from the Simplified Coping Style Questionnaire (SCSQ) developed by Xie (1998) was used to measure the positive coping levels of flight students. This dimension contains 12 items, such as “trying to view problems from different angles” and “seeking advice from family and friends,” reflecting constructive coping strategies individuals employ when facing stress. A 4-point Likert scale is used (0 = never used, 3 = frequently used), with dimensional total scores ranging from 0 to 36 points. Higher scores indicate greater tendency to adopt positive coping strategies. This questionnaire has demonstrated good reliability and validity among Chinese populations and has been widely applied in stress and coping research. In this study, the Cronbach’s *α* coefficient for the positive coping dimension was 0.95. Confirmatory factor analysis results showed: RMR = 0.02, CFI = 0.94, NFI = 0.94, NNFI = 0.93, TLI = 0.93, IFI = 0.94, PGFI = 0.62, PNFI = 0.77, PCFI = 0.77, SRMR = 0.03.

(4) Basic Need Satisfaction in General Scale (BNSG-S)

The Basic Need Satisfaction in General Scale (BNSG-S), originally developed by Gagné (2003) and revised by Du et al. (2020) for the Chinese population, was used to assess the degree of basic psychological need satisfaction among flight students. Based on self-determination theory, the scale includes three dimensions: (AN) autonomy need (7 items), (CN) competence need (6 items), and (RN)relatedness need (8 items), totaling 21 items. A 7-point Likert scale is employed (1 = completely does not apply, 7 = completely applies), with some items reverse-scored. Total scores range from 21 to 147 points, with higher scores indicating greater satisfaction of basic psychological needs. In this study, the overall Cronbach’s *α* coefficient for the scale was 0.752. Although this value is slightly below 0.80, it remains well above the universally accepted threshold of 0.70 for psychometric acceptability in exploratory behavioral research, indicating adequate internal consistency. Confirmatory factor analysis supported the three-factor structure: PNFI = 0.65, PCFI = 0.66.

### 2.3 Statistical method

All data analyses in this study were completed using SPSS 26.0. Statistical significance was determined using conventional alpha levels: *p* ≥ 0.05 was considered non-significant, *p* < 0.05 was considered statistically significant, and *p* < 0.001 was considered highly statistically significant. All hypothesis testing employed an alpha level of 0.05. First, reliability and validity tests were conducted on measurement instruments including perceived social support, simplified coping styles, basic psychological need satisfaction, and internet addiction to assess the effectiveness of the measurement model. Second, Pearson correlation analysis was used to examine the correlational relationships among variables. Based on this foundation, PROCESS macro Model 6 was employed to construct and test the chain mediation model, exploring the internal mechanisms by which perceived social support influences internet addiction among flight students through simplified coping styles and basic psychological need satisfaction. The significance of mediation effects was assessed using the Bootstrap method (5,000 repeated samples) with 95% confidence intervals (CI), with effects considered significant when CI does not include 0.

## 3 Results

### 3.1 Measurement model validity testing and common method Bias testing

(1) Measurement Model Validity Testing.

To assess the validity of the measurement model, this study employed the following testing methods: The KMO value for scale data was 0.974, exceeding the critical value of 0.6, and Bartlett’s test of sphericity showed an approximate chi-square value of 79503.444 (df = 2016, *p* < 0.01), indicating significant common variance among variables and meeting the prerequisite conditions for factor analysis.

(2) Common Method Bias Testing.

Considering that data were collected through self-report methods, results may be affected by common method bias caused by participants adopting similar consistent response patterns to all items. This study employed Harman’s single-factor test to conduct exploratory factor analysis on all measurement items. Factor analysis extracted 6 factors with eigenvalues all greater than 1, with the first factor explaining 18.79% of the variance after rotation, well below the 40% judgment threshold, indicating that common method bias had minimal impact on data collected through questionnaire surveys.

### 3.2 Demographic characteristics

This study analyzed data from 1,235 students at the Civil Aviation Flight University of China. Participants had a mean age of 18.80 ± 0.78 years. Regarding grade level, 727 were freshmen (58.87%) and 508 were sophomores (41.13%). For family residence, 1,048 were from urban areas (84.86%) and 187 from rural areas (15.14%). Regarding only-child status, 670 were only children (57.25%) and 565 were not only children (45.75%). For smoking or drinking habits, 10 participants (0.81%) only smoked cigarettes, 32 participants (2.59%) only drank alcohol, 33 (2.67%) had both habits, and 1,160 (93.93%) had neither habit (Table 1).

**Table 1:** Demographic characteristics of study participants.

Characteristics	Category	Total (*n* = 1,235)
Age (years)		18.80 ± 0.78
Grade *n* (%)	Freshman	727 (58.87%)
	Sophomore	508 (41.13%)
Family residence *n* (%)	Urban	1,048 (84.86%)
	Rural	187 (15.14%)
Only child *n* (%)	Only child	670 (54.25%)
	Not only child	565 (45.75%)
Smoking or drinking habits *n* (%)	Smoking only	10 (0.81%)
	Drinking only	32 (2.59%)
	Both	33 (2.67%)
	Neither	1,160 (93.93%)

Data were described as *n* (%) or mean ± SD.

### 3.3 Descriptive statistics

This study conducted descriptive statistical analysis on variables including internet addiction and its dimensions, basic psychological needs and its dimensions, positive coping strategies, and perceived social support and its dimensions. The results are shown in Table 2.

**Table 2:** Descriptive statistical analysis.

Variable	*N*	Mean	SD
IA (Internet addiction)	1,235	28.54	9.73
BPN (Basic psychological needs)	1,235	99.27	12.22
AN (Autonomy need)	1,235	32.90	4.97
CN (Competence need)	1,235	33.91	5.05
RN (Relatedness need)	1,235	32.46	5.96
PCS (Positive coping strategies)	1,235	28.20	6.85
PSS (Perceived social support)	1,235	73.55	10.89
FS (Family support)	1,235	24.49	3.73
FrS (Friend support)	1,235	24.38	3.89
SOS (Significant other support)	1,235	24.69	3.69

Data were described as *n* (%) or mean ± SD.

The total score for internet addiction (IA) was 28.54 ± 9.73, which is below the clinical cutoff score of 53 points, indicating that the research subjects generally exhibited relatively low levels of internet addiction. However, this still warrants attention, as even subclinical levels of internet use may impact the daily functioning and training performance of flight students.

The value of basic psychological needs (BPN) was 99.27 ± 12.22, suggesting a moderately high level of basic psychological need satisfaction among the flight students. Specifically, competence need (CN) scored highest (33.91 ± 5.05), followed by autonomy need (AN) (32.90 ± 4.97), and relatedness need (RN) (32.46 ± 5.96). This indicates that among the three basic psychological needs, flight students experienced the greatest satisfaction in their competence need, likely reflecting the structured training environment and clear performance feedback in flight training programs. The relatively balanced scores across the three dimensions suggest that the aviation training system provides relatively comprehensive support for students’ psychological needs.

The value of positive coping strategies (PCS) was 28.20 ± 6.85, which is at a medium-high level, indicating that when facing stress and difficulties, the research subjects tend to adopt relatively constructive coping methods. This characteristic may be closely related to the rigorous selection criteria and systematic training processes for flight students, as individuals with stronger adaptive capacities are more likely to successfully navigate the demanding flight training environment.

The value of perceived social support (PSS) was 73.55 ± 10.89, representing a high level of perceived social support. Among its subscales, significant other support (SOS) scored highest (24.69 ± 3.69), followed by family support (FS) (24.49 ± 3.73), and friend support (FrS) (24.38 ± 3.89). The relatively balanced scores across the three dimensions indicate that flight students perceive comprehensive social support from multiple sources, which may serve as an important protective factor for their mental health and training adaptation.

Overall, the sample of civil aviation flight students in this study exhibited low levels of internet addiction, moderately high levels of basic psychological need satisfaction, medium-high levels of positive coping ability, and high levels of perceived social support. These characteristics may reflect the combined effects of the rigorous selection process, structured training environment, and comprehensive support systems in civil aviation education. The relatively positive psychological profile suggests that most flight students possess adequate psychological resources to cope with the challenges inherent in flight training, though continued attention to internet use patterns and their potential impact on training outcomes remains warranted.

### 3.4 Pearson correlation analysis

**Figure 2:**
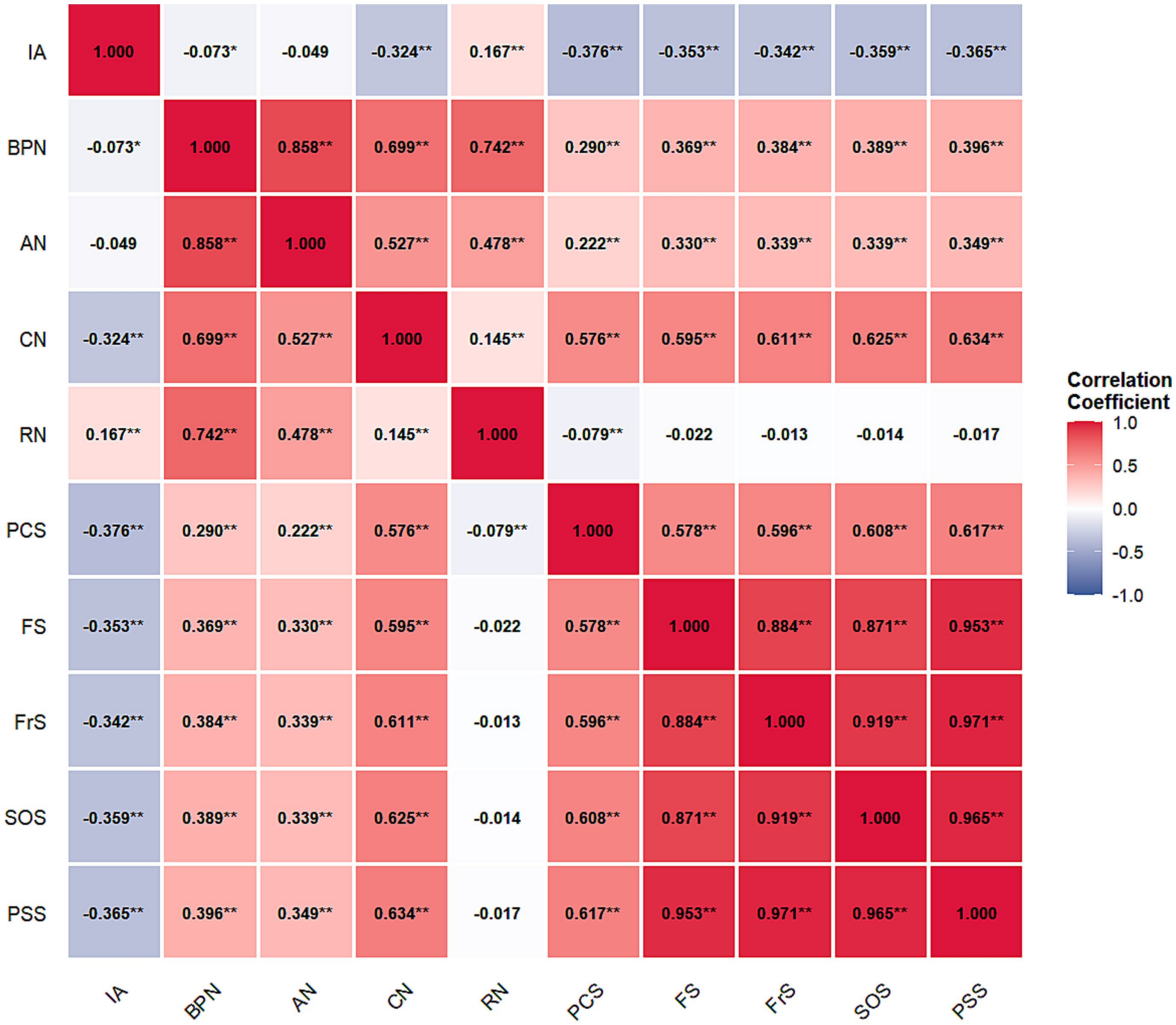
Pearson correlation coefficient result. IA, internet addiction; BPN, basic psychological needs; AN, autonomy need; CN, competence need; RN, relatedness need; PCS, positive coping strategies; PSS, perceived social support; FS, family support; FrS, friend support; SOS, significant other support. The color gradient represents correlation strength: blue indicates negative correlations, white indicates near-zero correlations, and red indicates positive correlations. Numbers in each cell show correlation coefficients with significance levels. *p < 0.05, **p < 0.01.

This study conducted Pearson correlation analysis on each variable (Figure 2). The results showed high positive correlations among basic psychological needs (BPN) and its dimensions: autonomy need (AN), competence need (CN), and relatedness need (RN) (*r* = 0.48–0.86, *p* < 0.01). Similarly, perceived social support (PSS) and its three dimensions demonstrated strong intercorrelations (*r* = 0.87–0.99, *p* < 0.01).

Internet addiction (IA) was significantly negatively correlated with basic psychological needs (*r* = −0.07, *p* < 0.05) and its dimensions, except autonomy need (*r* = −0.05, *p* > 0.05), with competence need showing the strongest negative correlation (*r* = −0.32, *p* < 0.01). Internet addiction also exhibited significant negative correlations with positive coping strategies (PCS) (*r* = −0.38, *p* < 0.01) and perceived social support and its dimensions (*r* = −0.34 to −0.37, *p* < 0.01). Notably, relatedness need showed a weak positive correlation with internet addiction (*r* = 0.17, *p* < 0.01).

Basic psychological needs and its dimensions demonstrated significant positive correlations with positive coping strategies (*r* = 0.22–0.58, *p* < 0.01), with competence need showing the strongest association (*r* = 0.57, *p* < 0.01). However, relatedness need was negatively correlated with positive coping strategies (*r* = −0.08, *p* < 0.01). Basic psychological needs and its dimensions (except relatedness need) were significantly positively correlated with perceived social support (*r* = 0.33–0.63, *p* < 0.01), with competence need demonstrating the strongest correlations (*r* = 0.60–0.63, *p* < 0.01).

Positive coping strategies exhibited strong positive correlations with perceived social support and all its dimensions (*r* = 0.58–0.62, *p* < 0.01). The correlation analysis indicated significant relationships among the main variables, providing a basis for subsequent chain mediation effect analysis.

### 3.5 Chain mediation model

This study tested the chain mediation model using Process 3.3 macro Model 6 in SPSS 26.0, while incorporating demographic variables (such as family residence and only-child status) as covariates for control. Mediation analysis results are shown in Table 3. After controlling for demographic variables, perceived social support had a significant negative predictive effect on internet addiction (*B* = −0.36, *p* < 0.01), supporting hypothesis H1. This indicates that perceived social support serves as a protective factor against internet addiction, with individuals perceiving high levels of social support showing fewer internet addiction symptoms. Additionally, perceived social support had significant positive predictive effects on positive coping strategies (*B* = 0.61, *p* < 0.01) and basic psychological need satisfaction (*B* = 0.35, *p* < 0.01), supporting hypotheses H2 and H3. This suggests that perceived social support helps individuals develop positive coping strategies and promotes satisfaction of their basic psychological needs. Further exploration of mediation pathways revealed: Positive coping strategies positively predicted basic psychological need satisfaction (*B* = 0.08, *p* < 0.01), supporting hypothesis H4. This indicates that individuals using positive strategies to cope with stress and challenges facilitates satisfaction of psychological needs such as autonomy, competence, and relatedness. These results preliminarily verified the chain mediation role of positive coping strategies and basic psychological need satisfaction between perceived social support and internet addiction.

**Table 3:** Regression results and bootstrap mediation effect analysis (*n* = 1,235).

Path	Std. β (Effect)	*t*-value	*p*-value	Boot SE	Boot LLCI	Boot ULCI
Direct effect						
Perceived Social Support → Internet Addiction	−0.25	−7.21	<0.01	0.03	−0.31	−0.18
Indirect effects						
Perceived Social Support → Positive Coping	0.61	27.28	<0.01	0.02	0.57	0.66
Perceived Social Support → Basic Needs	0.35	10.53	<0.01	0.03	0.29	0.42
Positive Coping → Basic Needs	0.08	2.25	0.03	0.03	0.01	0.14
Positive Coping → Internet Addiction	−0.25	−7.51	<0.01	0.03	−0.31	−0.18
Basic Needs → Internet Addiction	0.1	3.5	<0.01	0.03	0.04	0.15
Total effect						
Perceived Social Support → Internet Addiction	−0.36	−13.54	<0.01	0.03	−0.41	−0.31

Bootstrap 95% confidence intervals based on 5,000 resamples. Effects are significant when confidence intervals exclude zero. Control variables including family residence, only-child status, and smoking/drinking habits were included in the models but omitted from the table for brevity.

To further verify the robustness of the mediation effects, this study conducted mediation analysis using the Bootstrap method. As shown in Table 3, the total indirect effect of perceived social support on internet addiction through positive coping strategies and basic psychological need satisfaction was −0.113, accounting for 31.4% of the total effect. Among these, the mediation effect of positive coping strategies was −0.15 [95% CI = (−0.20, −0.11)]; the mediation effect of basic psychological need satisfaction was 0.03 [95% CI = (0.01, 0.06)]. Bootstrap 95% confidence intervals did not include 0, indicating that both mediation pathways were significant. Notably, the mediation effect of perceived social support through positive coping strategies was relatively stronger.

**Figure 3:**
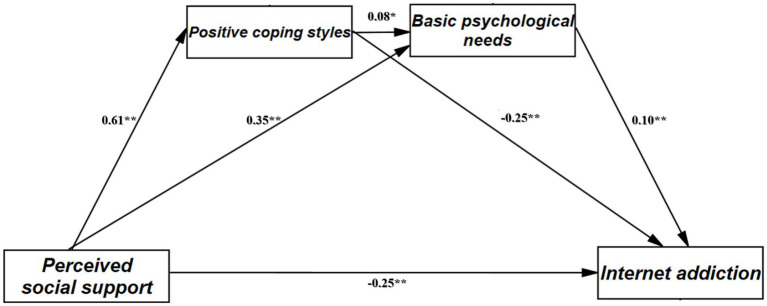
Chain mediation model of perceived social support on internet addiction. **p < 0.01.

In summary, positive coping strategies and basic psychological needs play a chain mediation role in the process by which perceived social support influences internet addiction, supporting the research hypotheses, as shown in Figure 3. The confidence intervals for total mediation effect, direct effect, and indirect effects did not include 0, indicating significance at the 0.05 level. This means that individuals who perceive more social support can not only directly reduce internet addiction symptoms but also indirectly avoid excessive dependence on the internet through mediation mechanisms of actively coping with setbacks and adversity, as well as obtaining satisfaction of psychological needs such as sense of belonging and competence.

## 4 Discussion

### 4.1 Direct effects of perceived social support on internet addiction among flight students

This study found that perceived social support significantly negatively predicted internet addiction among flight students (*B* = −0.36, *p* < 0.01), validating hypothesis H1. This result underscores that social support serves as a crucial protective factor against the risk of internet addiction within this specialized group. When positive coping strategies and basic psychological need satisfaction were included as mediating variables, the direct effect of perceived social support on internet addiction remained significant (*B* = −0.25, *p* < 0.01), though its magnitude was reduced. This indicates a partial mediation pattern, where perceived social support not only directly alleviates internet addiction but also indirectly influences it by activating internal psychological resources. This finding aligns with the social support buffering hypothesis, suggesting that social support operates through both direct and indirect pathways to impact mental health outcomes.

According to the social support buffering hypothesis, perceived social support operates through both main effects and buffering effects (Cohen and Wills, 1985). Research on Chinese adolescents during COVID-19 in 2020 found that social support not only directly negatively correlated with internet addiction but also indirectly influenced internet addiction through multiple mediations of resilience, PTSD symptoms, and their combination (Cui and Chi, 2021). This finding may hold special significance in the flight student population. Flight students face unique occupational pressures. From a social compensation theory perspective (Cheng et al., 2019), the enclosed management environment of flight students creates a distinctive social ecology. Compared to ordinary university students, flight students have more limited social circles, primarily confined to peer students, flight instructors, and family members. This relatively closed social environment makes every bit of social support particularly valuable. When students perceive adequate social support, their sense of belonging and worth are satisfied, reducing motivation to seek compensatory social interaction through the internet. Conversely, students lacking social support may view cyberspace as a refuge for escaping real-world pressures and seeking emotional comfort (Wu et al., 2016). The 2024 FAA Mental Health and Aeromedical Certification Aviation Rulemaking Committee report emphasized that lack of trust, fear, and perceived stigma are reasons why pilots do not report mental health issues (Cross et al., 2024). More importantly, different sources of social support play differentiated roles in the flight student population (Van de Casteele et al., 2024). Peer support is particularly crucial, as fellow students share experiences of high-intensity training and elimination pressure, potentially forming unique “comradeship.” Their understanding and support, based on shared experiences, are more targeted and effective. Support from flight instructors carries professional guidance qualities, providing not only emotional support but also teaching professional skills for coping with flight pressure. Family support, though physically distant, is maintained through regular video calls and provides important emotional backing. Research found that the synergistic effect of these three support sources forms a multi-layered protective network, effectively reducing internet addiction risk (Zhao et al., 2021). Additionally, qualitative research on Chinese civil aviation pilots found that pressure from assessment and medical qualification requirements creates heavy psychological burdens, particularly for older pilots (Xu et al., 2024). These occupation-specific pressures make the role of social support systems even more critical. Similarly, the FAA continues to emphasize providing mental health resources for pilots, including medical examiner training, research and clinical studies, and employing more mental health professionals to expand internal resources.

The emergence of partial mediation effects has important theoretical significance. Because the direct effect remains significant (*B* = −0.25), it indicates that perceived social support does not operate exclusively through internal psychological mechanisms (positive coping and need satisfaction); rather, social support itself also exerts a strong, independent buffering effect against internet addiction. This finding suggests that merely providing external support is insufficient; the key lies in helping flight students transform external support into internal psychological resources and adaptive behavioral patterns.

### 4.2 Mediation mechanism of positive coping strategies

Research confirmed that positive coping strategies significantly mediate the relationship between perceived social support and internet addiction (Effect = −0.15, 95% CI = [−0.20, −0.11]), supporting hypothesis H2. This mediation pathway accounted for approximately 41.7% of the total effect, highlighting its role as the most substantial indirect pathway in the model. This strong mediation effect in the flight student population may not be coincidental but closely related to their occupational characteristics and training environment.

According to transactional theory, flight students face unique stresses: high standards (zero tolerance for error), high intensity (daily flight training), and high uncertainty (potential elimination at any time). This high-pressure environment requires students to develop effective coping strategies to survive and thrive (Chen and Wu, 2022). Research found that perceived social support promotes the formation and application of positive coping strategies through multiple pathways (Cattelino et al., 2021). First, social support provides coping resources; for example, informational support may help students learn effective stress management techniques, while emotional support may enhance coping confidence (Acoba, 2024). Second, social support creates a safe psychological space, enabling students to dare to try new coping strategies without worrying about failure consequences. Finally, role modeling effects—seeing other students or instructors successfully cope with stress—enhance motivation to adopt similar strategies (Smith, 2021). In specific flight training contexts, positive coping strategies manifest across multiple levels. Cognitive aspects include positive cognitive restructuring (viewing flight challenges as growth opportunities) and reasonable attribution (attributing training mistakes to technical unfamiliarity rather than lack of ability) (Curtiss et al., 2021); behavioral aspects include actively seeking guidance from flight instructors, exchanging flight experiences with peer students, and developing detailed study plans; emotion regulation aspects include exercise for stress relief, meditation for relaxation, and humor for diffusion (Alkhawaldeh et al., 2023). Existing research shows that the comprehensive application of these positive coping strategies significantly reduces internet addiction risk. In contrast, students who adopt negative coping (such as avoidance, denial, self-blame) are more likely to temporarily forget stress through internet addiction (Yildirim Demirdöğen et al., 2024). Additionally, the importance of positive coping in the flight student population is further confirmed. Stress management can be as simple as regular exercise, reasonable diet, attention to alcohol intake, adequate rest, and spending time with friends and family—these steps can cultivate healthy lifestyles and help positively cope with routine stress (Ziaka et al., 2025). More importantly, a longitudinal study found that military personnel who reported more depressive symptoms at baseline reported less talking with friends and family and less exercise or sports as coping behaviors at follow-up (Morgan et al., 2017). As another specialized occupation, this indirectly supports the findings of this study.

### 4.3 Mediation mechanism of basic psychological need satisfaction

Basic psychological need satisfaction played a significant mediating role between perceived social support and internet addiction (Effect = 0.03, 95% CI = [0.01, 0.06]), validating hypothesis H3. Although this mediation effect was weaker than that of positive coping strategies, its mechanism of action may be more profound, perhaps involving the activation of individuals’ intrinsic motivation systems.

From a self-determination theory perspective, flight students’ basic psychological need satisfaction may face unique challenges. Regarding autonomy needs, quasi-military management and standardized training procedures seem to severely compress personal autonomous space (Lepinoy et al., 2021). Research findings suggest that perceived social support may partially alleviate this dilemma by providing psychological autonomy support—when instructors and peers understand and acknowledge students’ personal feelings, even in an environment with objectively limited autonomy, students may still experience some degree of autonomy (Slemp et al., 2024). This “psychological autonomy” may not change objective limitations but may be sufficient to maintain basic autonomy need satisfaction. Competence need satisfaction may show polarization in the flight student population. The high elimination rate of flight training makes competence feelings perhaps a scarce resource. Continuous competence anxiety may drive some students to seek alternative competence experiences through online games. Research findings suggest that perceived social support may help students establish progressive competence feelings by providing timely positive feedback and technical guidance (Hew et al., 2023). Professional recognition from flight instructors, in particular, may effectively buffer competence threats from training failures. Relatedness needs may be the most special among the three basic needs. Flight students’ enclosed training environment may create high-intensity but limited interpersonal interactions. Research findings suggest that when students perceive genuine social support, their sense of belonging may be satisfied, thereby reducing the need to seek virtual belonging through online social interaction. Establishing peer relationship cognition as a “learning community” rather than “competitors” may significantly improve relatedness need satisfaction levels.

The asymmetry between need satisfaction and need frustration may be worth attention (Olafsen et al., 2021). Research suggests that the negative impact of basic psychological need frustration may far exceed the positive impact of need satisfaction. This may explain why some students who receive adequate social support may still develop internet addiction—critical frustrating events may trigger compensatory internet use. Therefore, preventing need frustration may be more important than promoting need satisfaction.

### 4.4 Interpretation of the positive indirect effect and suppression effect

A counterintuitive finding in this study is that relatedness need showed a weak positive correlation with internet addiction (*r* = 0.17), and the independent mediation effect of basic psychological need satisfaction was positive (Effect = 0.03), despite the total and direct effects being significantly negative. This statistical pattern indicates a classical suppression effect. Methodologically, the Bootstrap analysis confirms the stability of this positive indirect pathway (Effect = 0.03, 95% CI = [0.01, 0.06]), indicating a robust structural relationship rather than a statistical artifact. In the highly regulated and enclosed environment of aviation training, offline social opportunities are severely limited. Consequently, flight students often rely on online multiplayer games or social media platforms to maintain peer relationships and team cohesion. Therefore, students with higher relatedness satisfaction may inherently have higher frequencies of digital engagement, which reflects on the internet addiction scale. Additionally, an alternative explanation is that students with higher psychological need satisfaction might generally possess greater social engagement and more opportunities for online interaction. This suggests that while basic psychological need satisfaction generally promotes well-being, the specific digitized means of achieving this connection within a restricted campus might inadvertently increase internet use duration.

Furthermore, while the independent mediation effect size of basic psychological need satisfaction is relatively small (0.03) compared to positive coping strategies (−0.15), it provides crucial theoretical evidence that basic psychological needs act as an active, albeit secondary, gear in the mechanistic chain between external support and addictive behaviors.

### 4.5 Integrated mechanism of chain mediation effects

The most important finding of this study is the significant chain mediation effect of positive coping strategies and basic psychological need satisfaction, validating hypothesis H4. This reveals the specific pathway where perceived social support influences positive coping strategies, which in turn enhances basic psychological need satisfaction, ultimately affecting internet addiction. The total indirect effect of the model was −0.113, and together, all mediation pathways (including the two independent paths and this chain path) accounted for 31.4% of the total effect of perceived social support on internet addiction. This comprehensive model potentially reveals the key psychological transmission mechanism from external social support to internal resources and finally to behavioral outcomes.

From the Job Demands-Resources model perspective, perceived social support and positive coping strategies may serve as resources that jointly address the high demands of flight training (Mazzetti et al., 2023). When resources are sufficient, they may not only cope with external demands but also create surplus for psychological need satisfaction. The high explanation rate of 94.0% suggests that this dynamic process may largely determine internet addiction risk among flight students. Compared to other theoretical models, the chain mediation model may demonstrate unique advantages. Parallel mediation models assume factors operate independently, potentially overlooking intrinsic connections; whereas chain mediation models may more clearly present the cascading process of influence. This study found good fit for the chain model through model comparison, suggesting the rationality of theoretical construction. Additionally, this chain pathway accounts for 17.8% of the total effect, revealing the cascading psychological process from social support to internet addiction. The chain mediation model is supported by related research. Studies indicate that future expectations may influence internet addiction through independent mediation effects of positive coping and basic psychological needs as well as their chain mediation effect (Du and Lyu, 2021). A study of Chinese rural high school students found that internet addiction negatively correlated with quality of life, with social support playing a mediating role in this association (Xiao et al., 2022). In specialized occupational groups, this chain effect is more prominent. A systematic review of active military personnel found that emotion regulation significantly affects decision-making, teamwork, stress management, and resilience, with effective emotion regulation being crucial for performance and mental health in high-pressure occupations (such as military, emergency services, and competitive sports) (Kirkham et al., 2025). Research on cognitive resilience in military personnel indicates that coping is not an automatic response to stressors but an effortful process that evolves as the nature of person-environment encounters changes, supporting the temporal characteristics of chain mediation (Flood and Keegan, 2022).

### 4.6 Research limitations and future directions

While achieving certain theoretical and practical contributions, this study has several limitations. First, this study employed a cross-sectional design, which can only reveal associations among variables and cannot determine causal relationships. Although the theoretically constructed chain mediation model has certain logical validity, the causal relationships among perceived social support, positive coping strategies, basic psychological need satisfaction, and internet addiction still require longitudinal studies for further verification. Particularly in the specialized population of flight students, psychological states may dynamically adjust with changes in training phases, assessment pressures, and career development, and single time-point measurements may not adequately capture this dynamic change process. Second, this study relied entirely on self-report questionnaires for data collection, which may involve social desirability bias and common method bias issues. The occupational pressures and image management needs faced by flight students may lead them to provide socially desirable responses when reporting sensitive topics like internet addiction. Regarding sample representativeness, this study’s sample came only from the Civil Aviation Flight University of China, with limited regional and institutional representation, and all participants were male students with relatively concentrated age distribution, mainly first- and second-year students. While this exclusively male sample accurately reflects the current demographic reality of Chinese civil aviation training programs, it limits the generalizability of our findings. Future research must investigate whether this chain mediation model operates differently among female pilots. This institution trains the vast majority of Chinese civil aviation pilots, its specific cultural context and highly collectivistic management style may limit the generalizability of these findings to Western or other international aviation training systems (e.g., FAA or EASA frameworks). Future research should examine similar flight student populations globally to cross-culturally validate these results. In terms of measurement instruments, although the scales used in this study have good reliability and validity among Chinese populations, these tools were primarily developed and validated for general university student populations, and their applicability to the specialized occupational group of flight students remains to be tested. Additionally, the internet addiction scale could not distinguish different types of internet addiction behaviors. Finally, although this study constructed a relatively complete chain mediation model, it may still have omitted other important variables such as sleep quality, academic pressure, personality traits, etc., and was primarily based on Western psychological theories with insufficient consideration of specificities within the Chinese cultural context.

Based on these limitations, future research should improve and expand in multiple aspects. In research design, longitudinal designs are recommended, using multi-time-point tracking surveys to more accurately reveal causal relationships and dynamic change processes among perceived social support, positive coping strategies, basic psychological need satisfaction, and internet addiction, spanning the complete training cycle of flight students to comprehensively understand psychological factor changes across different training phases. Multiple data collection methods should be combined, such as experience sampling for real-time data collection, physiological indicator measurements to assess stress states, behavioral observations to record internet use behaviors, and in-depth interviews to understand subjective experiences, forming a more comprehensive and objective data foundation. In sample selection, the sample scope should be expanded to include multiple flight academies, different regions, different genders of flight students, with particular attention to female pilot populations, and comparative studies with other high-risk occupational groups should be conducted to improve research generalizability and identify the uniqueness of flight student populations. In measurement tool development, specialized measurement instruments should be developed or revised based on flight student population characteristics, professional stress assessment tools should be developed for their unique stressors, and more refined classification measurement tools should be developed to distinguish different types of internet addiction behaviors. Theoretical model expansion should incorporate more possible mediating and moderating variables such as emotion regulation strategies, cognitive flexibility, psychological capital, etc., while considering environmental and cultural factor influences and exploring more complex statistical models such as multilevel linear models and latent class analysis. Additionally, conducting cross-cultural research is important for forming more universal theoretical models, and combining emerging technological approaches such as big data analysis, virtual reality technology, and artificial intelligence will bring innovation to research methods. In practical applications, research findings can guide flight academies in constructing multi-level internet addiction prevention and intervention systems, optimizing mental health services, reforming educational training models, providing scientific evidence for civil aviation management departments to formulate relevant policies, and supporting the development of intelligent mental health monitoring and intervention platforms to achieve early identification and timely intervention, providing more comprehensive and effective scientific guidance for mental health promotion in the specialized population of flight students.

## 5 Conclusion

Through a survey of 1,235 male students from the Civil Aviation Flight University of China, explored the mechanisms underlying the relationship between perceived social support and internet addiction. Research results indicate: (1) Perceived social support was significantly and negatively associated with internet addiction levels among flight students (*B* = −0.36, *p* < 0.01), validating the protective role of social support; (2) Both positive coping strategies and basic psychological need satisfaction played significant mediating roles between perceived social support and internet addiction. Positive coping strategies exerted a negative mediating effect (Effect = −0.15), while basic psychological need satisfaction showed a positive mediating effect (Effect = 0.03); (3) More importantly, a significant chain mediation pathway exists where positive coping strategies influence basic psychological need satisfaction. The total indirect effect was −0.113, and all mediation pathways combined accounted for 31.4% of the total effect of perceived social support on internet addiction, revealing a cascading transmission mechanism from external support to internal psychological resources to behavioral outcomes. However, it is crucial to delineate the boundary conditions of this study. The findings presented herein are particularly applicable to the highly controlled, quasi-military environments characteristic of civil aviation flight training. Due to the unique enclosed social ecology, rigorous elimination rates, and specific occupational stressors faced by flight students, caution should be exercised when generalizing these results to general university populations or other occupational groups that do not operate under similar environmental constraints.

Based on these research findings, this study proposes the following targeted recommendations: First, construct a specialized, stigma-free social support network system for flight students. Given the direct protective role of perceived social support, flight academies should establish a “trinity” support system including peer mutual assistance groups, flight instructor mentorship systems, and regular school-family communication mechanisms, particularly strengthening support density during high-pressure training periods and assessment phases through institutional arrangements to ensure every student can timely perceive and obtain support resources from different sources. Second, integrate positive coping skills training into the flight student cultivation system, such as embedding specific “digital wellness and stress management” modules into early-stage flight simulator training. Considering the strong mediation role of positive coping strategies, mandatory stress management courses should be offered to systematically teach positive coping strategies such as problem-solving, cognitive restructuring, and seeking support, and through flight simulator scenario training and case discussions, help students skillfully apply these strategies in actual flight pressure situations. Third, optimize the training environment to satisfy basic psychological needs through psychological empowerment. Recognizing the autonomy constraints inherent in quasi-military management, academies should implement structured empowerment initiatives. For instance, allowing student committees to co-design non-flight daily schedules or introducing peer-leadership rotations can provide micro-level autonomy allowances that effectively alleviate institutional constraints. Additionally, establishing progressive skill certification systems can continuously provide competence feedback, and regularly organize team-building activities and provide diverse offline recreational facilities to strengthen sense of belonging, thereby reducing students’ motivation to seek compensatory satisfaction through the internet, students’ motivation to seek compensatory satisfaction through the internet. Fourth, establish chain intervention mechanisms to prevent internet addiction. Based on chain mediation effect findings, design a “support-coping-needs” three-level coordinated intervention plan: first identify high-risk students with insufficient social support through regular psychological assessments, then conduct targeted coping skills workshops to enhance their positive coping abilities, and finally ensure basic psychological needs are met through personalized psychological counseling, forming a complete prevention and control chain from source prevention to process intervention to outcome assurance, minimizing internet addiction risk among flight students.

## Data Availability

The original contributions presented in the study are included in the article/Supplementary material, further inquiries can be directed to the corresponding author.
